# HSP90 Inhibitor, AT13387 Mitigates Chronic Lung Injury in Pre-Pubertal Mice: A Therapeutic Axis in Bronchopulmonary Dysplasia

**DOI:** 10.3390/biom16091320

**Published:** 2026-09-11

**Authors:** Pavel A. Solopov, Christiana Dimitropoulou, John D. Catravas, Ruben M. L. Colunga Biancatelli

**Affiliations:** 1 Frank Reidy Research Center for Bioelectrics, Old Dominion University, Norfolk, VA 23509, USA; psolopov@odu.edu (P.A.S.); cdimitro@odu.edu (C.D.); jcatrava@odu.edu (J.D.C.); 2 School of Medical Diagnostic & Translational Sciences, Ellmer College of Health Sciences, Old Dominion University, Norfolk, VA 23509, USA; 3 Department of Internal Medicine, Division of Pulmonary and Critical Care Medicine, Eastern Virginia Medical School, Macon and Joan Brock Virginia Health Sciences at Old Dominion University, Norfolk, VA 23508, USA

**Keywords:** hydrochloric acid, AT13387, onalespib, HSP90, pulmonary fibrosis, children, pre-pubertal, NLRP3, TGF-β, chronic lung injury, bronchopulmonary dysplasia, snRNA-Seq

## Abstract

Exposure to hydrochloric acid (HCl) can provoke severe chronic pulmonary injury. Children are particularly vulnerable due to their smaller airways, higher respiratory rate, and stronger inflammatory response; yet no countermeasures exist for HCl-induced chronic lung injury in the pediatric population. We have previously demonstrated the involvement of HSP90 during HCl-induced lung injury in pre-pubertal (p24) mice, who develop stronger persistent inflammation with higher NLRP3 inflammasome activation, but less pulmonary fibrosis compared to adults. Here, we tested the hypothesis that post-treatment with the second-generation HSP90 inhibitor AT13387 (Onalespib), administered subcutaneously beginning 24 h after HCl instillation, would prevent HCl-induced chronic lung injury and pulmonary fibrosis in young (p24) C57BL/6J mice. Pre-pubertal (p24) C57BL/6J mice received a single intratracheal instillation of 0.1 N HCl and were treated with AT13387 (10 mg/kg, s.c., 3×/week for 30 days) beginning 24 h post-exposure. Bronchoalveolar lavage fluid (BALF) analysis, lung function measurements, histological assessment (Ashcroft fibrosis score), and Western blot analysis of key signaling mediators were performed. Additionally, publicly available single-nucleus RNA sequencing (snRNA-Seq) data from a cohort of 13 infants with bronchopulmonary dysplasia (BPD) and 11 age-matched controls were analyzed to evaluate HSP90 isoform expression in human pediatric lung disease. AT13387 significantly reduced BALF white blood cell concentration without affecting total BALF protein levels. Importantly, AT13387 did not impair normal weight gain or development over the 30-day observation period. AT13387 abrogated HCl-induced expression of TGF-β1, phosphorylation of HSP90, ERK1/2, SMAD2, IκBα, and upregulation of the inflammasome NLRP3. Additionally, AT13387 prevented changes in lung function dynamics and reduced the Ashcroft fibrosis score. Analysis of the human snRNA-Seq BPD dataset revealed a widespread pan-overexpression of all three HSP90 isoforms, i.e., HSP90AA1, HSP90AB1, and HSP90B1. These findings suggest that the HSP90 inhibitor AT13387 exhibits strong antidotal properties against HCl-induced chronic lung injury and pulmonary fibrosis in a pre-clinical pediatric model and identify HSP90 as a conserved therapeutic target in pediatric chronic lung disease.

## 1. Introduction

HSP90 is a ubiquitous molecular chaperone comprising 1–2% of the total cellular proteome that participates in the proper folding and stabilization of over 400 client proteins [1]. HSP90 promotes NF-κB nuclear translocation and activates the inflammasome NLRP3. HSP90 inhibitors preferentially bind the phosphorylated (activated) form of HSP90, expressed predominantly in inflamed tissues, with ~1000-fold higher affinity than the constitutive form, conferring a favorable therapeutic index [2]. Several HSP90 inhibitors have demonstrated anti-fibrotic properties in multiple organ systems [3,4,5], including hydrochloric acid (HCl)-induced pulmonary fibrosis in adult mice [6]. Among these, AT13387 (Onalespib) is a second-generation, non-ansamycin HSP90 inhibitor with high selectivity and a favorable safety profile [7]. Unlike earlier HSP90 inhibitors, AT13387 does not exhibit hepatotoxicity and has demonstrated tolerability in Phase I/II clinical trials in adults and, notably, has been tested in pediatric populations with solid tumors with an acceptable safety profile [8]. AT13387 can be administered subcutaneously, making it suitable for pre-hospital and resource-limited settings. We have previously shown that AT13387 blocked the development of HCl-induced pulmonary fibrosis in adult mice [9].

HCl is among the most widely produced hazardous chemicals worldwide, due to its usage in the steel industry, oil and gas drilling, science laboratories, and illicit drug manufacturing [10]. Its inhalation is considered one of the top 10% most dangerous chemical exposures to human health [11], especially for children, with the Department of Homeland Security Chemical Threat Risk Assessment listing HCl among the agents against which antidotes are actively sought by the NIH CounterACT program [12]. Accidental spills of HCl have occurred near schools and public facilities, resulting in the hospitalization of children in Japan, the United States, and elsewhere [13,14]. HCl gas is colorless but, upon contact with atmospheric moisture, forms dense white fumes that concentrate near ground level, placing smaller individuals at heightened risk. Children possess smaller airways, a greater lung surface area relative to body weight, and a faster respiratory rate. Consequently, the CDC estimates that children receive proportionally larger deposited doses of HCl and thus develop more severe injury [14]. A single inhalation has been associated with Acute Distress Respiratory Syndrome, chronic lung injury, and pulmonary fibrosis [6,15,16]. However, little is known about the long-term consequences of HCl exposure in children. In our previous work, we developed a pediatric murine model of HCl exposure utilizing pre-pubertal p24 mice and demonstrated that, compared with adults, young animals develop stronger persistent inflammation with higher NLRP3 inflammasome activation but display a lesser fibrotic response with primarily airway involvement [17]. Thus, we hypothesized that post-treatment with AT13387 would prevent HCl-induced chronic lung injury and pulmonary fibrosis in young (p24) mice without impairing their normal growth and development. Furthermore, to strengthen the clinical rationale for targeting HSP90 in pediatric lung disease, we interrogated publicly available snRNA-Seq data from a large cohort of infants with bronchopulmonary dysplasia (BPD) to determine whether HSP90 isoforms are overexpressed in the diseased pediatric lung.

## 2. Materials and Methods

### 2.1. Materials

HCl ACS grade, methacholine USP grade, RIPA buffer, and protease inhibitor cocktail were obtained from Sigma-Aldrich Corporation (St. Louis, MO, USA). AT13387 (onalespib) was obtained from Selleck Chemicals (Houston, TX, USA) and dissolved in warmed DMSO, then diluted in corn oil to a final DMSO concentration of no more than 2.5%. Socumb (pentobarbital) USP grade, Anased (xylazine) USP grade, and Ketaset (ketamine) USP grade were supplied by Henry Schein Animal Health (Pittsburg, PA, USA). Formaldehyde (10%) was purchased from Thermo Fisher Scientific (Waltham, MA, USA), the BCA Protein assay kit from Pierce Co. (Rockford, IL, USA), and EDTA and Western blot membranes from GE Healthcare (Chicago, IL, USA). TRIzol^®^ and SuperScript™ IV VILO reverse transcriptase kit were from Invitrogen (Carlsbad, CA, USA), RNeasy Mini Kit from Qiagen (Hilden, Germany), and SYBR Green Master Mix from Applied Biosystems (Carlsbad, CA, USA). All primers used for real-time quantitative PCR were purchased from Integrated DNA Technologies, Inc. (Coralville, IA, USA). For SDS-PAGE, ProtoGel (30% acrylamide mix) and TEMED were from National Diagnostics (Atlanta, GA, USA), Tris-HCl buffer from Teknova (Hollister, CA, USA), 10% SDS and ammonium persulfate from Thermo Fisher Scientific, and Protein Dual Color Standards and Tricine Sample Buffer from Bio-Rad Laboratories (Hercules, CA, USA).

All antibodies were purchased from reputable commercial sources with published immunospecificity data. For Western blots: rabbit anti-total and anti-phosphorylated ERK1/2 (#9102, #4370), HSP90 (#4874, #3488), NLRP3 (#15101), P-SMAD2 (#8828), SMAD2 (#5339), P-SMAD3 (#9520), SMAD3 (#9513), IκBα (#4814), and P-IκBα (#2859) were from Cell Signaling Technology, Inc. (Danvers, MA, USA); mouse monoclonal anti-beta actin was from Sigma-Aldrich Corporation; and IRDye 800CW Goat anti-rabbit and IRDye 680RD Goat anti-mouse antibodies were from LI-COR Biosciences (Lincoln, NE, USA).

### 2.2. Ethical Statement

All animal studies were approved by the Old Dominion University Institutional Animal Care and Use Committee (IACUC) and adhered to the principles of animal experimentation as published by the American Physiological Society. Studies were carried out in Biosafety Level 2 (BSL-2) and Animal Biosafety Level 2 (ABSL-2) facilities at the Frank Reidy Research Center for Bioelectrics in Norfolk, Virginia.

### 2.3. Animals and Experimental Design

Male C57BL/6J mice (Jackson Laboratories, Bar Harbor, ME, USA; stock #000664) of 21 days of age (p21) were housed under pathogen-free conditions with a 12-h dark/light cycle and ad libitum access to food and water. C57BL/6J mice represent an optimal wild-type model to study pulmonary inflammation. A two-day acclimation period was allowed before experimentation. For our experiments, the experimental unit was the individual mouse. At day 24, p24 mice, approximately 8–10 g in body weight corresponding to a human age of 6–9 years [18], were randomly divided into three treatment groups:

1. Saline group: Intratracheal instillation of sterile saline (2 µL/g body weight).
2. HCl group: Intratracheal instillation of 0.1 N HCl (2 µL/g body weight) + subcutaneous corn oil vehicle (3×/week starting 24 h after instillation for 30 days).
3. HCl + AT13387 group: Intratracheal instillation of 0.1 N HCl (2 µL/g body weight) + AT13387 (10 mg/kg in corn oil, s.c., 3×/week starting 24 h after instillation for 30 days). The 30-day endpoint was chosen because HCl-induced chronic lung injury and pulmonary fibrosis are fully established by day 30 in this model [16]. The 3×/week subcutaneous schedule was adapted from our previously validated adult AT13387 regimen [9] and reflects the durable pharmacodynamic target engagement of onalespib, which persists beyond its plasma half-life.

Sample size was estimated a priori using a power analysis for a one-way ANOVA with three groups. Based on previous studies using the HCl intratracheal instillation model in juvenile mice from our laboratory [17], the primary outcome measure (Ashcroft fibrosis score) was expected to show a mean difference (δ) of approximately 2.0 units between the HCl and Saline groups, with a pooled standard deviation (SD) of approximately 0.8. Using these parameters (effect size f = δ/(2 × SD) = 1.25; α = 0.05, power = 0.80, 3 groups), a minimum of n = 4 animals per group was required (G*Power v.3.1.9.7, Heinrich-Heine-Universität Düsseldorf, Germany). To account for potential attrition due to the severity of the model in young mice, we aimed for n = 5 per group for each outcome measure, yielding a total of 15 animals. A total of 45 male C57BL/6J mice were used in this study, allocated as follows: Saline group (n = 14), HCl group (n = 15), and HCl + AT13387 group (n = 16). Not all animals underwent every analysis. We planned 4–5 mice per group for BALF, 4–5 for Western Blotting, Flexivent, and RT-PCR; and 3–4 for Histology. Due to the small body size of these animals (8–10 g) and the smaller large airways and trachea, we observed a higher-than-expected attrition, with 2 mice in each group dying during the surgical and post-surgical period, reducing the numbers per group to Saline (n = 12), HCl (n = 13) and HCl + AT13387 (n = 14). Thus, within each group, we allocated n = 3–5 for WB, n = 4–5 for BALF and n = 3–4 for Histology. After survival surgeries, mice were closely monitored for signs of pain or distress. A standalone criterion for euthanasia was the loss of more than 25% of body weight. In case of distress, the veterinarian would be contacted for additional care or euthanasia, while in case of extreme loss of body weight, euthanasia would be performed. No mouse was removed from the study after the first 24 h of surgical and post-surgical period.

### 2.4. Intratracheal Instillation

On day 0, mice were anesthetized with intraperitoneal (i.p.) injections of ketamine (90 mg/kg) and xylazine (9 mg/kg). A pre-emptive i.p. bolus of sterile normal saline (10 µL/g) was administered for fluid resuscitation. A small neck skin incision (~1 cm) was made to visualize the trachea. Mice were suspended vertically from their incisors and a fine (25G) plastic catheter was advanced into the trachea (~1.5 cm). Freshly prepared 0.1 N HCl or sterile saline (2 µL/g body weight) was introduced through the catheter and flushed with 50 µL air. The catheter was withdrawn, the neck incision was closed with Vetbond surgical adhesive, and animals were placed over a heating pad with supplemental oxygen progressively weaned from 100% to 21%. Mice were observed for four hours for signs of respiratory distress before being returned to their cages.

### 2.5. HCl Dose Calculation

As we have previously described [17], 2 µL/g of 0.1 N HCl was instilled into p24 mice, corresponding to a 7.17 mg/kg dose. Using allometric calculations [18], the equivalent deposited human dose was computed to be 0.58 mg/kg for children, corresponding to a child of 20 kg exposed for 5 min to approximately 2000 ppm HCl, consistent with the “dangerous” exposure threshold [19].

### 2.6. BALF Analysis

BALF was obtained by instilling and withdrawing sterile 1× PBS (1 mL) via a tracheal cannula. The fluid was centrifuged at 2500× *g* for 10 min; the supernatant was stored at −80 °C for protein analysis. The cell pellet was re-suspended in 1 mL sterile PBS and total white blood cell (WBC) count was determined using a hemocytometer. Total protein concentration was quantified using a micro-BCA Protein Assay Kit.

### 2.7. Histopathology and Lung Fibrosis Scoring

Lungs were fixed by tracheal instillation of 10% formaldehyde at 15 cm H_2_O pressure, ligated, and placed in formaldehyde for 72 h. Mid-transverse slices were embedded in paraffin; 5-µm sections were stained with H&E and Masson’s trichrome. Pulmonary fibrosis was quantified using the method of Ashcroft et al. (1988) [20]. For each animal, ten randomly selected non-overlapping fields per slide were examined at 100× magnification, and each field was graded on a scale from 0 to 8 as follows: 0 = normal lung; 1 = minimal fibrous thickening of the alveolar or bronchiolar walls; 2–3 = moderate thickening of the walls without obvious damage to lung architecture; 4–5 = increased fibrosis with definite damage to lung structure and formation of fibrous bands or small fibrous masses; 6–7 = severe distortion of structure and large fibrous areas (“honeycomb lung” placed in this category); 8 = total fibrous obliteration of the field. Each field was assessed at low magnification and assigned the grade corresponding to the most severe degree of fibrosis present: the mean of all field scores was calculated to yield a single fibrosis score per animal. The observer was blinded to the treatment group.

### 2.8. Western Blot Analysis

Proteins were extracted from frozen lung tissue by sonication (50% amplitude, 3 × 10 s) in ice-cold RIPA buffer with protease inhibitor cocktail (100:1). After centrifugation at 14,000× *g* for 10 min, supernatants were collected and total protein concentration was determined by micro-BCA assay. Equal amounts of protein were separated on 10–12% polyacrylamide SDS gels, transferred to nitrocellulose membranes, and probed with appropriate primary antibodies followed by fluorescent secondary antibodies. Detection was performed using a LI-COR Odyssey CLx imaging system. Bands were quantified by densitometry using ImageJ v.1.8.0 (NIH, Bethesda, MD, USA), normalized to β-actin, and expressed as the ratio of phosphorylated to total protein. Uncropped full membranes are provided as Supplemental Data.

### 2.9. RNA Isolation and Quantitative Real-Time PCR

Lung tissue was homogenized in TRIzol^®^, followed by purification using the RNeasy Mini Kit. Purified RNA was transcribed into cDNA using SuperScript™ IV VILO reverse transcriptase and analyzed by real-time qPCR with SYBR Green Master Mix on a StepOne Plus Real-Time PCR System (Applied Biosystems v.2.3). Results were evaluated using the standard curve method and expressed as fold of control, normalized to β-actin. Primer pairs for Fibronectin (Fibronectin F: GAA GTC GCA AGG AAA CAA GC; Fibronectin R: GTT GTA GGT GAA CGG GAG GA), collagen 1α2 (Collagen 1α2 F: GAA GCA CGT CTG GTT TGG A; Collagen 1α2 R: ACT CGA ACG GGA ATC CAT C), TGF-β1 (TGF-b1 F: 5′-GGA CGA GCT GGT TGA GAG AA-3′; TGF-b1 R: 5′-GCA GTA GCA GCG GAA AAG TC-3′) and β-actin (Beta actin F: 5′-CCC CTG AGG AGC ACC GTG TG-3′; Beta-actin R: 5′-ATG GCT GGG GTG TTG AAG GT-3′) were employed as previously described [9].

### 2.10. Lung Mechanics Measurements

Mice were anesthetized with pentobarbital (90 mg/kg, i.p.), tracheostomized with a metal 1.2 mm cannula, and connected to a FlexiVent small animal ventilator (SCIREQ Inc., Montreal, QC, Canada). Ventilation was performed at a tidal volume of 10 mL/kg and respiratory rate of 150/min with a 15-min stabilization period. Pressure–volume (PV) loops were measured by stepwise increasing airway pressure to 30 cm H_2_O. Snapshot-150 and Quick Prime-3 maneuvers yielded respiratory system resistance (Rrs), Newtonian resistance (Rn), elastance (Ers), tissue damping (G), and tissue elastance (H). Methacholine challenge was performed with aerosolized methacholine at concentrations from 0 to 50 mg/mL, with Rrs, Rn, and Ers recorded at each concentration.

### 2.11. Single-Nucleus RNA Sequencing (snRNA-Seq) Analysis of the Human BPD Cell Atlas

Publicly available snRNA-Seq data were obtained from the human BPD cell atlas generated by Sun and Pryhuber [21], accessible at https://app.lungmap.net/app/shinycell-bpd (accessed on 15 June 2026). This dataset comprises snRNA-Seq performed on lung tissue from 13 infants with BPD and 11 age-matched controls who died from complications associated with prematurity between 4 months and 3 years of age. Unsupervised clustering identified over 30 distinct cell populations spanning epithelial, mesenchymal, endothelial, and immune compartments. Expression of three HSP90 isoforms, HSP90AA1 (stress-inducible cytoplasmic), HSP90AB1 (constitutive cytoplasmic), and HSP90B1 (ER-resident/GRP94) was mapped onto UMAP embeddings and visualized using the integrated ShinyCell browser provided by the atlas.

### 2.12. Statistical Analysis

Results are expressed as means ± standard error of the mean (SEM). Statistical significance was determined by one-way or two-way ANOVA followed by Tukey’s post-hoc or Bonferroni’s test, using GraphPad Prism Software v10.4.1 (Dotmatics, San Diego, CA, USA). The significance level was set at *p* < 0.05.

## 3. Results

### 3.1. AT13387 Reduces HCl-Induced Alveolar Inflammation Without Affecting Weight Gain

**Figure 1:**
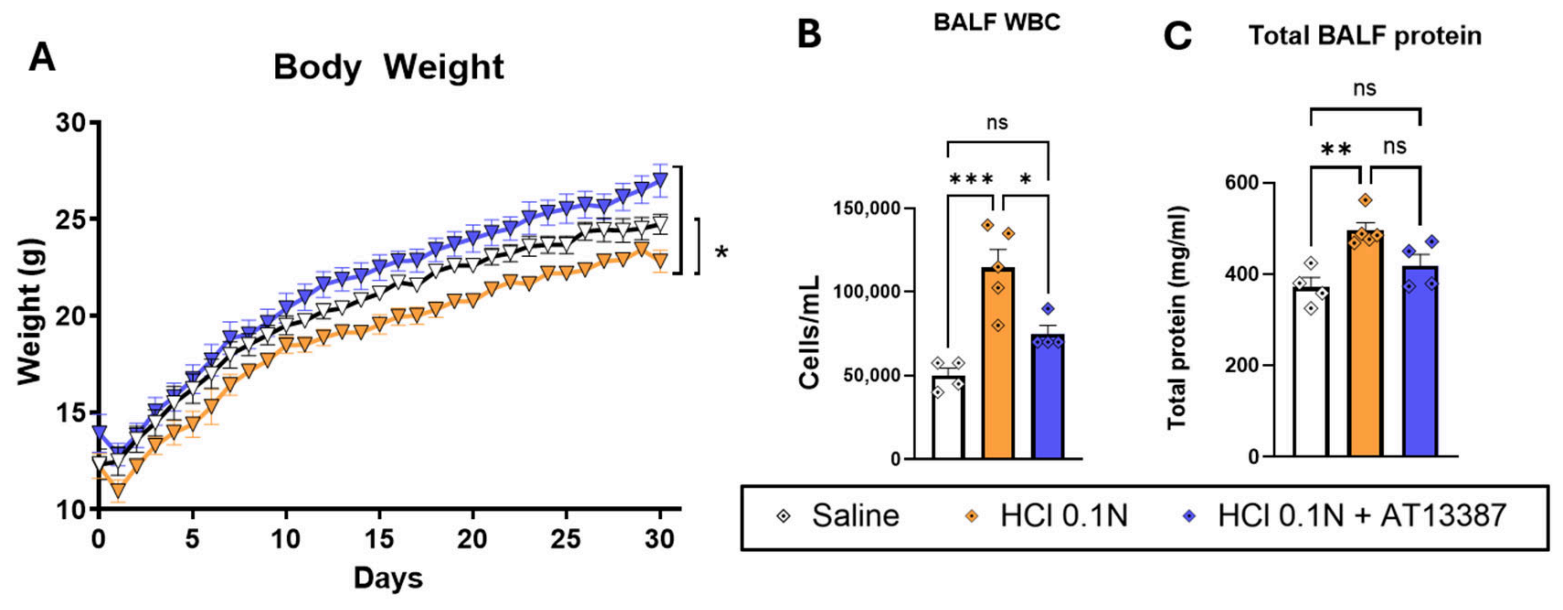
AT13387 reduces HCl-induced alveolar inflammation and preserves weight gain in prepubescent mice. (**A**) Daily body weight monitoring over 30 days. (**B**) Total BALF WBC count, and (**C**) BALF protein concentration 30 days after intratracheal instillation of saline, HCl, or HCl + 10 mg/kg AT13387 3x/week s.c. Data ± SEM, n = 4–5 group; * *p* < 0.05; ** *p* < 0.01; *** *p* < 0.001, ns = not significant, with 2-way ANOVA and Bonferroni’s posttest (1A), and 1-way ANOVA and Tukey’s post-test (**B**,**C**).

Following intratracheal instillation of 0.1 N HCl, p24 mice were monitored daily for 30 days. HCl-instilled mice showed a transient reduction in weight gain during the first 5–10 days, which became significantly different from saline controls by day 13, and remained significant till day 30 (Figure 1A; *p* < 0.01). Importantly, post-treatment with AT13387 (10 mg/kg, s.c., 3×/week starting 24 h after HCl instillation) reduced weight loss and weight trajectories comparable to the saline group throughout the 30-day period. The dose of AT13387 was replicated from previous studies in adult mice [9]. Notably, mice in the AT13387 group had slightly higher initial weights compared to saline or HCl groups. BALF analysis on day 30 revealed that HCl instillation provoked a persistent increase in total WBC counts (*p* < 0.001 vs. saline) and total protein concentration (*p* < 0.01 vs. saline). AT13387 significantly reduced BALF WBC counts (*p* < 0.05 vs. HCl alone), while total BALF protein did not reach statistical significance between HCl and HCl + AT13387 groups (Figure 1B,C).

### 3.2. AT13387 Abrogates HCl-Induced Activation of Pro-Fibrotic and Inflammatory Signaling Pathways

**Figure 2:**
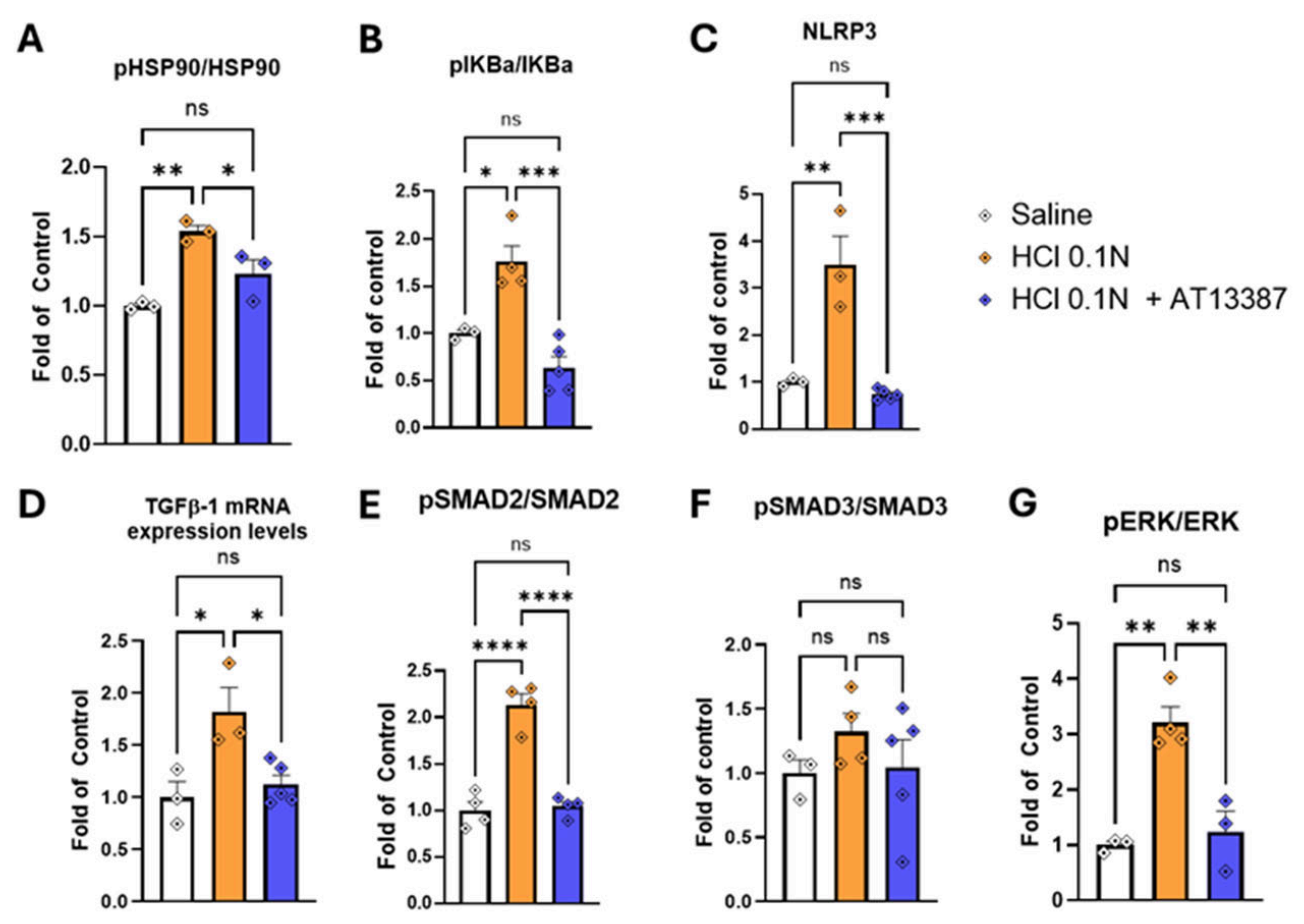
AT13387 abrogates HCl-induced activation of pro-fibrotic and inflammatory signaling in lung homogenates of prepubescent mice. Western blot densitometry and RT-PCR quantification of (**A**) P-HSP90, (**B**), p-IKBα, (**C**) NLRP3, (**D**) TGF-β1 mRNA, (**E**) pSMAD2/SMAD2, (**F**) pSMAD3/SMAD3, and (**G**) p-ERK. Data ± SEM, n = 3–5 group; * *p* < 0.05; ** *p* < 0.01; *** *p* < 0.001; **** *p* < 0.0001, ns = not significant, with 1-way ANOVA and Tukey’s post-test.

To investigate the molecular mechanisms underlying the protective effects of AT13387, we measured the expression and activation of key signaling mediators in lung homogenates 30 days after HCl instillation. HCl induced a significant increase in the phosphorylated (activated) form of HSP90 (*p* < 0.01 vs. saline), which was effectively reversed by AT13387 (*p* < 0.05 vs. HCl), confirming target engagement in vivo (Figure 2A). The NF-κB pathway, assessed by IκBα phosphorylation, was activated by HCl (*p* < 0.05 vs. saline) and completely blocked by AT13387 (*p* < 0.001 vs. HCl), consistent with the known HSP90-dependent regulation of NF-κB nuclear translocation [22]. Similarly, AT13387 was able to modulate the consequent activation of the inflammasome NLRP3 (Figure 2C, *p* < 0.01). HCl instillation significantly increased TGF-β1 mRNA and both the canonical SMAD2/SMAD3 and non-canonical ERK signaling. AT13387 was able to modulate both signaling pathways as previously published (Figure 2D–G, *p* < 0.05).

### 3.3. AT13387 Prevents HCl-Induced Chronic Lung Dysfunction

**Figure 3:**
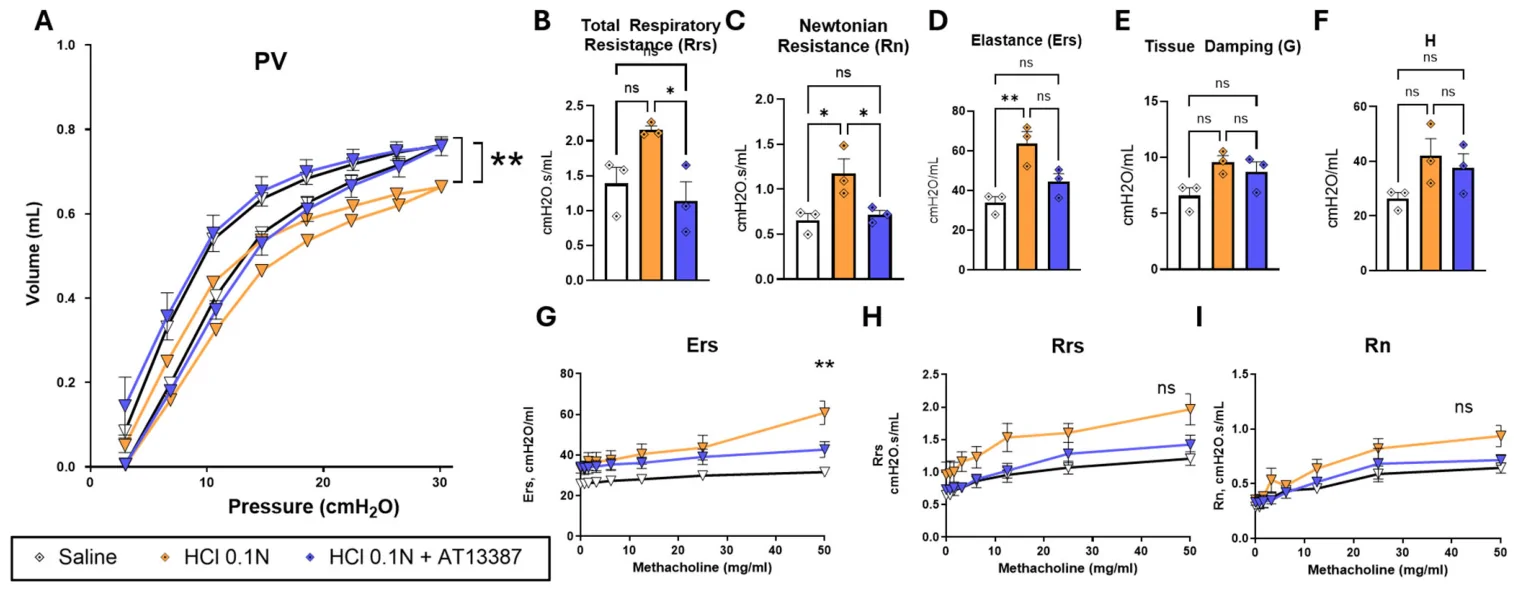
AT13387 prevents HCl-induced chronic lung dysfunction in prepubescent mice. (**A**) PV loops and baseline. (**B**) Total respiratory resistance (Rrs), (**C**) Newtonian resistance (Rn), (**D**) elastance (Ers), (**E**) tissue damping (G), (**F**) tissue elastance, (H) lung parameters measured via Flexivent. Methacholine challenge was performed with increasing doses of methacholine (0–50 mg/mL) for (**G**) elastance, (**H**) total respiratory resistance, and (**I**) Newtonian resistance. Data ± SEM, n = 3 per group; * *p* < 0.05; ** *p* < 0.01; ns = not significant, with 1-way or 2-way ANOVA and Tukey’s post-test.

Lung mechanics were assessed using the FlexiVent system 30 days after HCl instillation (Figure 3). HCl-instilled p24 mice displayed a significant downward shift in PV loops compared with saline controls (*p* < 0.01), reflecting stiffer, less compliant lungs (Figure 3A). Total and Newtonian resistances, with the latter reflecting resistance of the large conducting airways, were significantly increased in HCl-treated young mice (*p* < 0.01 vs. saline), consistent with our prior observation that young mice develop primarily airway involvement (Figure 3B,C). Elastance increased under basal conditions and in response to methacholine, suggesting that HCl increased bronchial hyperresponsiveness (Figure 3D,G).

Treatment with the HSP90 inhibitor not only improved the downward shift in PV loops but also prevented the shift in resistances and minimized the HCl increase in elastance. AT13387 was not able to reduce the HCl-induced changes in tissue damping or tissue elastance (H) (Figure 3E,F).

### 3.4. AT13387 Reduces HCl-Induced Pulmonary Fibrosis

**Figure 4:**
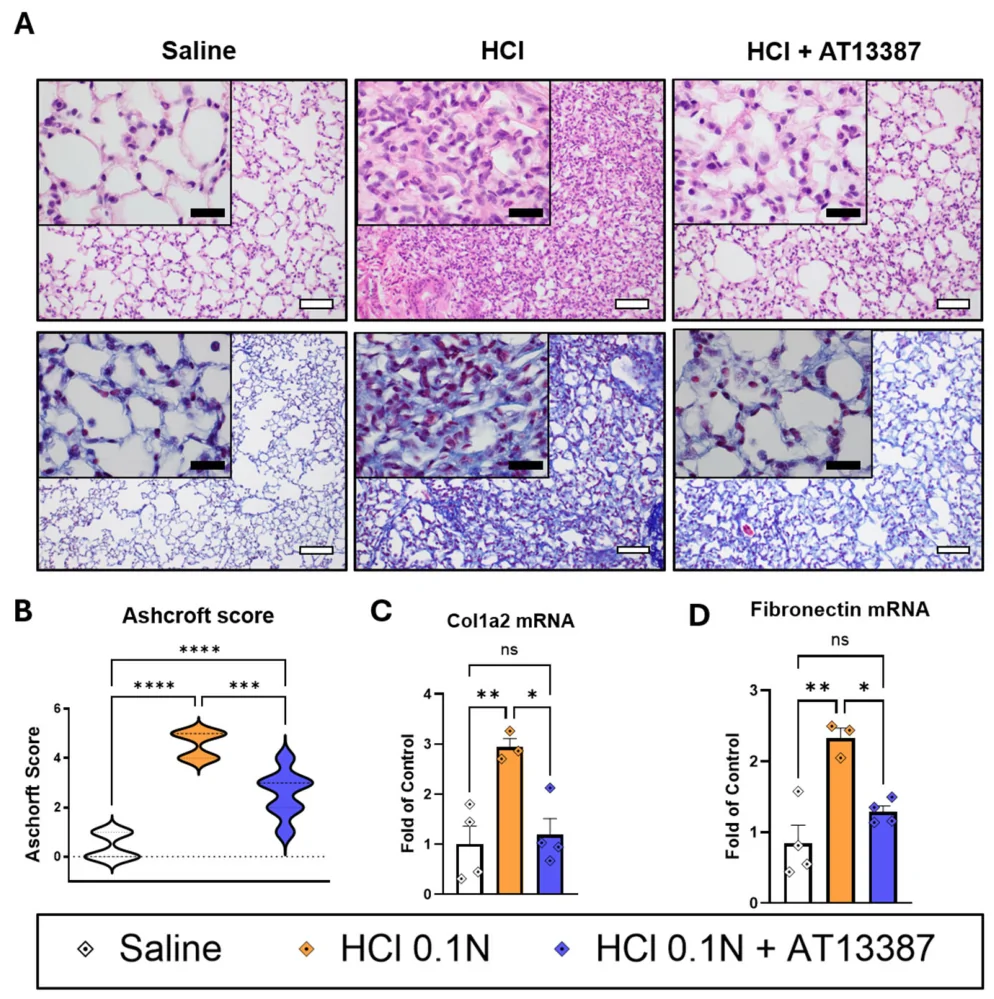
AT13387 attenuates HCl-induced pulmonary fibrosis in prepubescent mice. (**A**) Representative lung slides stained for H&E (**top**) and Masson’s trichrome (**bottom**) for 100× and 10× with black bars corresponding to 10 µm and white bars to 50 µm. Lung injury was quantified by the Ashcroft fibrosis score (**B**). RNA extracted from lungs of mice was quantified by RT-PCR for (**C**) collagen 1α2 mRNA, and (**D**) fibronectin mRNA. Histology representatives for Masson’s trichrome for saline and HCl groups were previously compared and published to adult mice exposed to saline or HCl [17]. Data ± SEM, n = 3–4 per group; * *p* < 0.05; ** *p* < 0.01; *** *p* < 0.001, **** *p* < 0.0001, ns = not significant, with 1-way ANOVA and Tukey’s post-test.

Histological analysis of lungs harvested at day 30 was performed using H&E and Masson’s trichrome staining. Lungs from HCl-instilled p24 mice showed increased cellularity, thickening of alveolar septa, mononuclear cell infiltration, and areas of collagen deposition. AT13387-treated lungs displayed markedly improved architecture, with preserved alveolar structures and substantially less collagen deposition, resembling the saline controls (Figure 4A, top). The Ashcroft fibrosis score was significantly elevated in HCl-instilled young mice compared with saline controls (*p* < 0.001). AT13387 treatment reduced the Ashcroft score (*p* < 0.001 vs. HCl), although values remained modestly higher than those of saline controls, reflecting some residual remodeling (Figure 4A bottom, Figure 4B).

HCl significantly increased collagen 1α2 mRNA expression and fibronectin compared to saline, with treatment of the HSP90 inhibitor improving both biomarkers of ECM deposition (Figure 4C,D).

### 3.5. HSP90 Isoforms Are Overexpressed Across All Cell Populations in the Lungs of Infants with Bronchopulmonary Dysplasia

**Figure 5:**
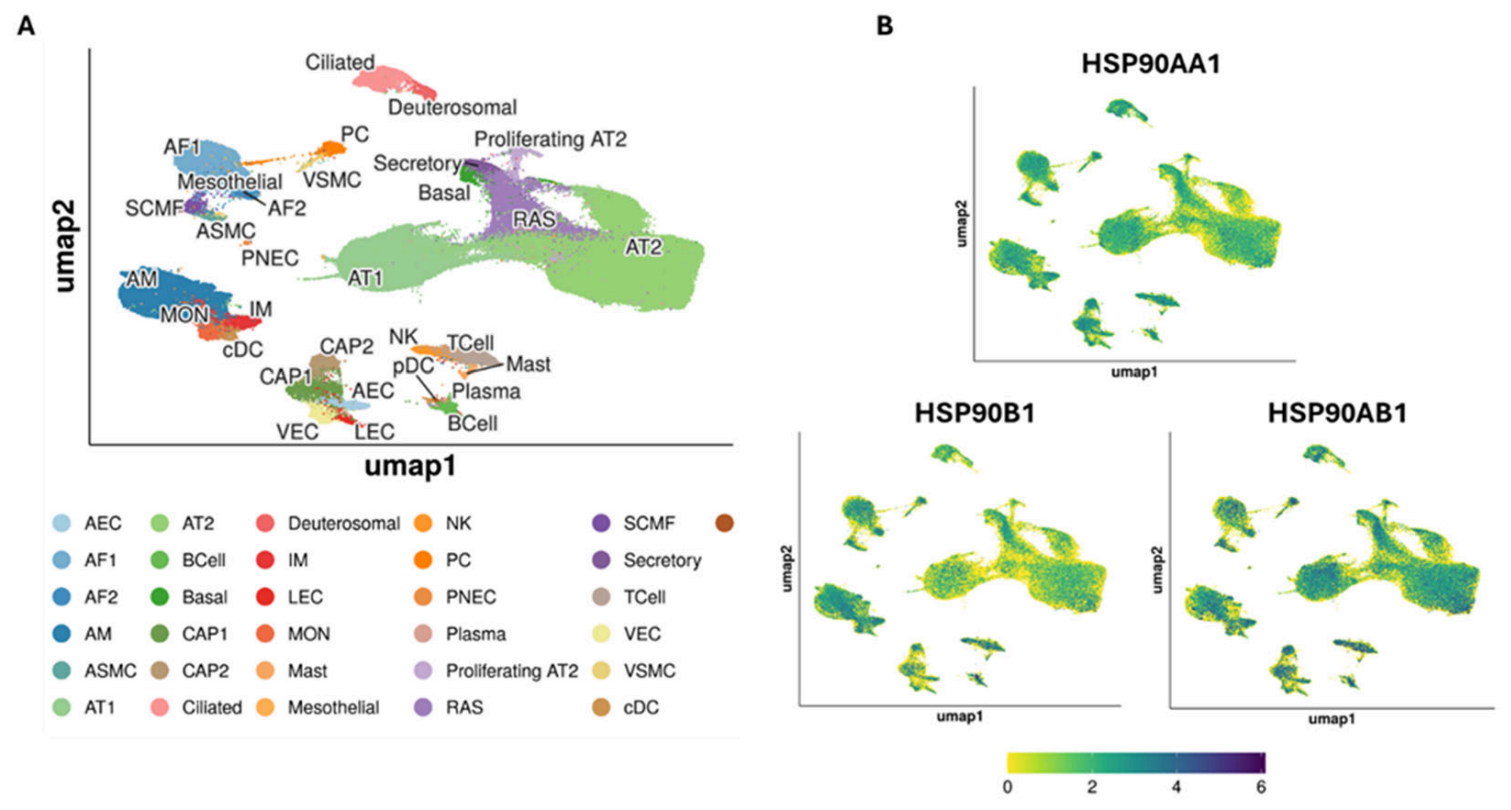
All three HSP90 isoforms are ubiquitously overexpressed across all cell populations in the lungs of infants with BPD. snRNA-Seq data from Sun and Pryhuber [21]. (**A**) UMAP embedding colored by cell population identity. (**B**) Three panels showing UMAP embeddings colored by expression of HSP90AA1, HSP90AB1, and HSP90B1. Dataset available at: https://app.lungmap.net/app/shinycell-bpd (accessed on 15 June 2026).

To determine whether HSP90 overexpression is a feature of human pediatric chronic lung disease, we interrogated a recently published human BPD single-nucleus RNA sequencing (snRNA-Seq) cell atlas generated by Sun and Pryhuber [21]. This atlas comprises snRNA-Seq data from lung tissue of 13 infants with BPD and 11 age-matched controls who died between 4 months and 3 years of age from complications associated with prematurity. Unsupervised clustering identified over 30 distinct cell populations spanning epithelial cells (AT1, AT2, proliferating AT2, basal, secretory, ciliated, deuterosomal, PNEC, recently described airway secretory [RAS] cells), mesenchymal cells (ASMC, VSMC, pericytes, adventitial fibroblasts [AF1, AF2], SCMF, mesothelial cells), endothelial cells (aerocytes, general capillary [CAP1, CAP2], venous, lymphatic), and immune cells (alveolar macrophages, interstitial macrophages, monocytes, cDC, pDC, T cells, B cells, NK cells, mast cells, plasma cells) (Figure 5A). UMAP expression mapping of all three major HSP90 isoforms-HSP90AA1 (stress-inducible cytoplasmic), HSP90AB1 (constitutive cytoplasmic), and HSP90B1 (ER-resident/GRP94 [23])—revealed widespread, high-level expression across virtually every identified cell population in the BPD lungs (Figure 5B). HSP90AA1 showed particularly robust expression distributed across all epithelial, mesenchymal, endothelial, and immune clusters, with intense signal in AT2 cells, RAS cells, alveolar macrophages, monocytes, and proliferating AT2 cells. HSP90B1 displayed a similarly broad distribution pattern with notably a strong signal in secretory cells, AT2 cells, proliferating AT2 cells, and endothelial populations. HSP90AB1 also showed ubiquitous expression, with the strongest signal in immune cell clusters and epithelial populations.

The overexpression of all three HSP90 isoforms across the entire cellular landscape of BPD lungs indicates a profound, global activation of the HSP90 chaperone system in this disease. This pan-isoform, pan-cellular overexpression pattern is consistent with the widespread cellular stress, active TGF-β signaling, NF-κB activation, and fibroproliferative remodeling that characterize BPD [24,25], and provides a strong molecular rationale for the therapeutic application of HSP90 inhibitors in this vulnerable population.

## 4. Discussion

**Figure 6:**
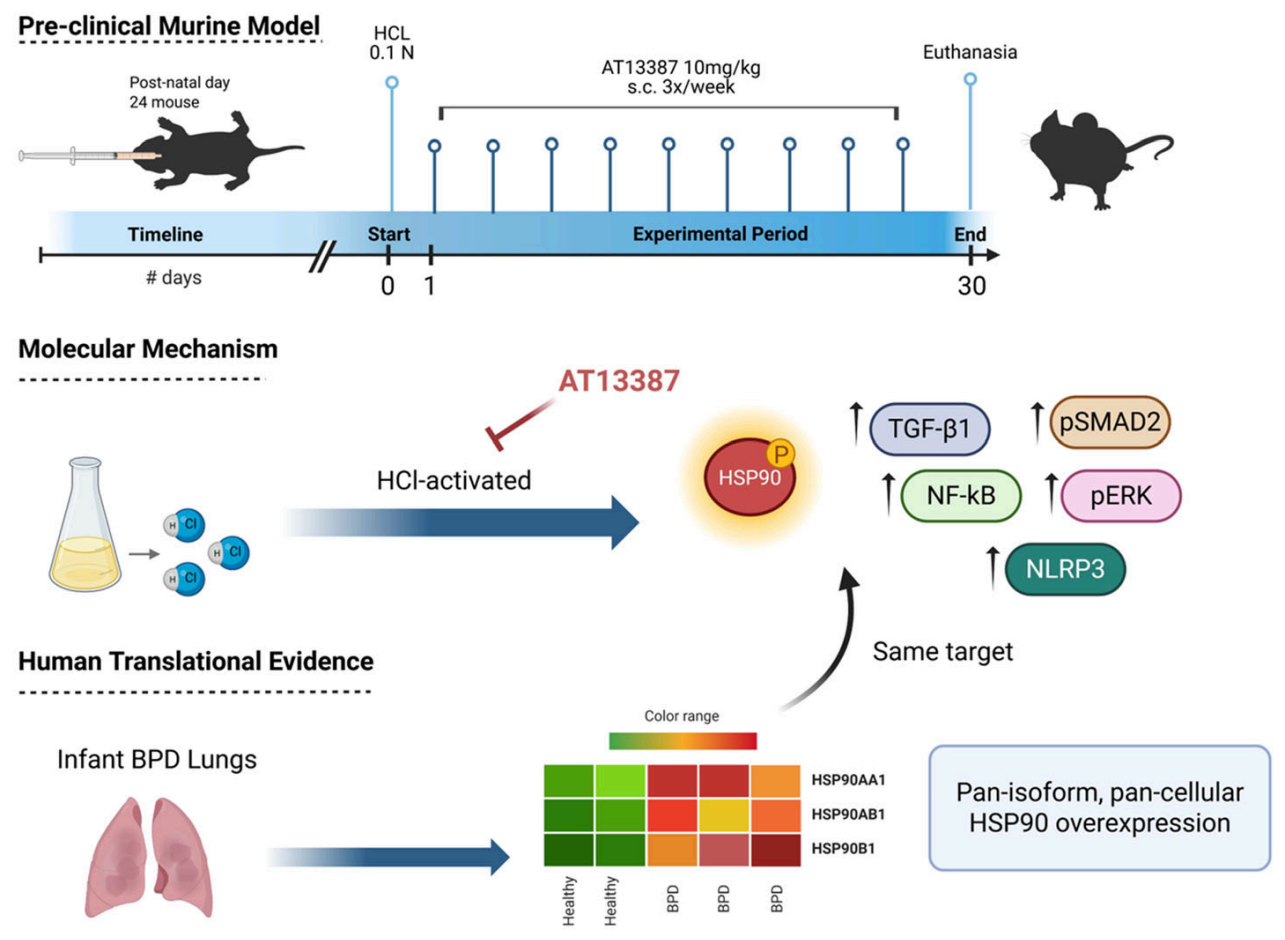
AT13387 prevents HCl-induced chronic lung injury in pre-pubertal mice by targeting the pHSP90 signaling hub. (**Top Panel**) Pre-pubertal (p24) C57BL/6J mice received intratracheal HCl (0.1 N) on Day 0 followed by AT13387 (10 mg/kg, s.c., 3×/week) from Day 1 through Day 30. (**Central Panel**) AT13387 inhibits phosphorylated HSP90 (pHSP90), thereby blocking downstream pro-fibrotic (TGF-β1/pSMAD2/pERK) and pro-inflammatory (NF-κB/pIκBα/NLRP3) cascades and reducing collagen and fibronectin deposition. (**Bottom Panel**) Single-nucleus RNA-Seq data from 13 infants with bronchopulmonary dysplasia (Sun and Pryhuber, 2024) confirm pan-isoform, pan-cellular overexpression of HSP90AA1, HSP90AB1, and HSP90B1, identifying HSP90 as a conserved therapeutic target in pediatric chronic lung disease (bottom tier). Created in BioRender.com (NF29UN8RMY).

In this study, we demonstrate that the HSP90 inhibitor AT13387 prevents HCl-induced chronic lung injury, inflammation, and pulmonary fibrosis in a pre-clinical pediatric murine model. Post-treatment with AT13387, initiated 24 h after a single intratracheal instillation of HCl, a clinically realistic intervention window comprehensively blocked the molecular, histological, and functional hallmarks of chronic lung disease in young (p24) mice without impairing their normal growth and development. Furthermore, we provide evidence that all three major HSP90 isoforms are ubiquitously overexpressed in the lungs of infants with BPD, a clinically analogous pediatric chronic lung disease, providing a translational rationale for HSP90 inhibition as a therapeutic strategy in pediatric chronic lung disease (Figure 6).

The rationale for targeting HSP90 in HCl-induced chronic lung injury stems from the central role of this chaperone in sustaining multiple pathways that drive inflammation and fibrosis [6]. HSP90 stabilizes the TGF-β receptor and its downstream effectors ERK and SMAD2 [26], promotes NF-κB nuclear translocation [22], and activates the inflammasome NLRP3 [27]. By destabilizing these client proteins simultaneously, HSP90 inhibitors offer a uniquely comprehensive approach to blocking both the pro-fibrotic and pro-inflammatory cascades that perpetuate chronic lung injury. Our molecular data (Figure 2) demonstrate that AT13387 abrogated HCl-induced activation of TGF-β1, pERK, pSMAD2, pHSP90, pIκBα, and NLRP3. This multi-target inhibition is consistent with the mechanism of HSP90 inhibitors, which preferentially bind the activated (phosphorylated) form with ~1000-fold higher affinity than the constitutive form [2], thereby promoting proteasomal degradation of client proteins selectively in stressed tissues. A notable finding was the absence of significant SMAD3 phosphorylation in HCl-instilled p24 mice, in contrast to the robust pSMAD3 activation we have documented in adult mice [6]. This differential SMAD pathway engagement suggests that the relative contribution of canonical versus non-canonical TGF-β signaling differs between developing and mature lungs and may partly explain the more inflammatory and less purely fibrotic phenotype we previously observed in young mice [17].

The prevention of NLRP3 inflammasome activation by AT13387 is particularly relevant to the pediatric context. We previously demonstrated that young p24 mice develop significantly higher NLRP3 levels than adults following HCl exposure, suggesting that the inflammasome contributes disproportionately to chronic lung injury in the young. NLRP3 activation has been implicated in both asthma [28] and pulmonary fibrosis [29], and its inhibition may represent a dual therapeutic target. The near-complete suppression of NLRP3 by AT13387 (Figure 2C) suggests that young animals, who display a predominantly inflammatory response, may derive even greater benefit from HSP90 inhibition than adults. By disrupting the HSP90-NLRP3 interaction, AT13387 effectively blocks inflammasome-driven injury without the growth-suppressive effects associated with corticosteroids.

The lung mechanics data (Figure 3) further suggests the comprehensive protection afforded by AT13387. The normalization of PV loops, Rn, Rrs, and Ers indicates that AT13387 prevented both the restrictive pattern of fibrosis and the obstructive pattern of airway injury that characterize HCl-induced chronic lung dysfunction in young mice. The attenuation of methacholine hyperresponsiveness further suggests prevention of the airway remodeling and bronchial hyperreactivity that are hallmarks of post-exposure asthma-like conditions in children. The preservation of weight gain in AT13387-treated mice (Figure 1C) addresses a critical concern for any therapeutic intervention in the pediatric population. Chronic corticosteroid therapy, the current recommended treatment for toxic chemical inhalation in children, is known to impair growth and provoke metabolic complications [30]. Additionally, increased TGF-β levels can undermine post-natal lung development by reducing surfactant protein expression and inhibiting alveolar branch morphogenesis [31]. By normalizing TGF-β signaling without the systemic side effects of corticosteroids, AT13387 may offer a therapeutic advantage for children.

The snRNA-Seq analysis of BPD lungs (Figure 5) adds a critical translational dimension to this study. BPD is a chronic lung disease affecting premature infants, characterized by arrested alveolar and vascular development, persistent inflammation, and fibrotic remodeling. BPD remains the most common serious complication of preterm birth, affecting up to 40% of extremely low-birth-weight infants, and is associated with lifelong respiratory morbidity [32]. Critically, BPD pathogenesis is driven by dysregulation of the TGF-β axis: elevated TGF-β signaling inhibits alveolarization, impairs surfactant production, and drives mesenchymal expansion [33]. These are the same HSP90-dependent pathways that AT13387 comprehensively blocked in our murine model. The demonstration that all three HSP90 isoforms, HSP90AA1, HSP90AB1, and HSP90B1, are overexpressed across every identified cell population in BPD lungs indicates that HSP90 activation is not confined to a single pathogenic cell type but reflects a tissue-wide stress response. The robust expression of HSP90AA1 in macrophage and monocyte populations aligns with the known role of HSP90 in sustaining NF-κB signaling and NLRP3 inflammasome activation [27], both implicated in BPD pathogenesis [34]. The overexpression in mesenchymal populations is consistent with HSP90-mediated TGF-β receptor stabilization, which drives the mesenchymal expansion observed in BPD. The overexpression in AT2 and proliferating AT2 cells is particularly relevant, as AT2 cells are the progenitors of alveolar epithelial regeneration [35]; HSP90-driven TGF-β signaling in AT2 cells has been linked to impaired regeneration and promotion of epithelial–mesenchymal transition [26]. The involvement of HSP90B1 (GRP94), which chaperones secreted proteins including TGF-β itself, suggests that the ER stress response, implicated in BPD pathogenesis [36], may be an additional mechanism through which HSP90 inhibition could confer benefit. Despite being encouraging and interesting, these findings still rely on the comparison of two different disease models (HCl instillation and BPD) across two different species and different translated ages (mice equal to 6–9 years old, BPD in newborns). These limitations must be considered, particularly in regard to possible translated treatments with HSP90 inhibitors to the BPD population.

Indeed, the translational potential of HSP90 inhibitors is strengthened by several features of AT13387: it has been tested in pediatric clinical trials (NCT02535800) with an acceptable safety profile [8], substantially de-risking its repurposing; the subcutaneous route of administration is practical for pre-hospital settings; and the dose used (10 mg/kg s.c.) represents 30–50% of the dose we have employed in adult mice [9], selected to balance efficacy with tolerability in young animals.

Our study has several limitations. We investigated a single time-point (30 days) and a single HCl concentration (0.1 N); dose–response and time-course studies would further characterize the therapeutic window. A further limitation is that only male mice were studied. Because estrogen has been shown to regulate both HSP90 expression and NLRP3 inflammasome activity, the response of female animals may differ substantially. We did not compare AT13387 to corticosteroids, nor completed dedicated pharmacokinetic/pharmacodynamic characterization of AT13387 in pre-pubertal mice. Since AT13387 has been evaluated clinically, with known safety and toxicity, we decided not to include a Saline + AT13387 control arm. While we assessed overall growth by body weight monitoring, more detailed analyses of lung development, including mean alveolar linear intercept, surfactant protein expression, and branching morphogenesis markers, are needed to fully characterize the impact of AT13387 on the developing lung. Finally, the surgical attrition reduced our final group sizes to 3–5 animals, below the n = 5 targeted by our a priori power analysis, so the study was underpowered and non-significant comparisons should be interpreted with caution. Importantly, the effects that did reach significance did so despite this reduced power, suggesting the true effect sizes are large enough to survive a smaller sample, though the findings may benefit from confirmation in an adequately powered cohort.

The BPD snRNA-Seq atlas, although derived from a large cohort (n = 13 BPD, n = 11 controls) [21], was interrogated here for qualitative expression patterns rather than formal differential expression analysis; cell-type-specific quantitative comparisons are an important next step. The dataset does not include pre- or post-treatment conditions with HSP90 inhibitors, and functional validation requires dedicated studies. The comparison between p24 instilled with HCl and newborns with BPD relies on the strong lung inflammatory response.

## 5. Conclusions

This study demonstrates that the HSP90 inhibitor AT13387, administered subcutaneously starting 24 h after HCl instillation, prevents the development of chronic lung injury and pulmonary fibrosis in pre-pubertal (p24) mice. AT13387 comprehensively blocked HCl-induced TGF-β/ERK/SMAD signaling, NF-κB/IκBα activation, HSP90 phosphorylation, and NLRP3 inflammasome upregulation without impairing normal weight gain. The observation that all three HSP90 isoforms are ubiquitously overexpressed in the lungs of infants with BPD provides direct human translational evidence that HSP90 is a conserved therapeutic target in pediatric chronic lung disease. These findings support the further development of HSP90 inhibitors as countermeasures against HCl-induced chronic lung injury in children and, potentially, as a novel therapeutic strategy for BPD, a devastating condition for which no targeted pharmacological therapy currently exists.

## Data Availability

Data supporting the findings of this study are available from the corresponding author upon request. The snRNA-Seq BPD cell atlas is publicly available at https://app.lungmap.net/app/shinycell-bpd (accessed on 15 June 2026).

## Supplementary Materials

The following supporting information can be downloaded at: https://www.mdpi.com/article/10.3390/biom16091320/s1, Original Western blot membranes are attached as supplemental materials. In order of appearance, p-HSP90, HSP90, p-IKBa, IKBa, NLRP3, p-SMAD2, SMAD2, p-SMAD3, SMAD3, p-ERK, and ERK.

## Abbreviations

The following abbreviations are used in this manuscript:

HCl	Hydrochloric acid
HSP90	Heat Shock Protein 90
BALF	Bronchoalveolar lavage fluid
WBC	White blood cell
TGF-β	Transforming growth factor beta
NLRP3	NLR family pyrin domain-containing protein 3
ERK	Extracellular signal-regulated kinase
SMAD	Mothers against decapentaplegic homolog
NF-κB	Nuclear factor kappa B
IκBα	Inhibitor of nuclear factor kappa B alpha
BPD	Bronchopulmonary dysplasia
snRNA-Seq	Single-nucleus RNA sequencing
UMAP	Uniform manifold approximation and projection
AT1/AT2	Alveolar type 1/type 2 cells
ECM	Extracellular matrix
PV	Pressure-volume
Rrs	Respiratory system resistance
Rn	Newtonian resistance
Ers	Elastance of the respiratory system
H&E	Hematoxylin and eosin
qPCR	Quantitative polymerase chain reaction
IACUC	Institutional Animal Care and Use Committee
SEM	Standard error of the mean
ANOVA	Analysis of variance
