# Supplementary material for: HSP90 Inhibitor, AT13387 Mitigates Chronic Lung Injury in Pre-Pubertal Mice: A Therapeutic Axis in Bronchopulmonary Dysplasia
Source: Biomolecules. 2026 Sep 11;16(9):1320. doi: 10.3390/biom16091320 (PMC13604055; doi:10.3390/biom16091320)
Supplement: Supplementary file 1 [file biomolecules-16-01320-s001.zip › biomolecules-4459152-supplementary.pdf]

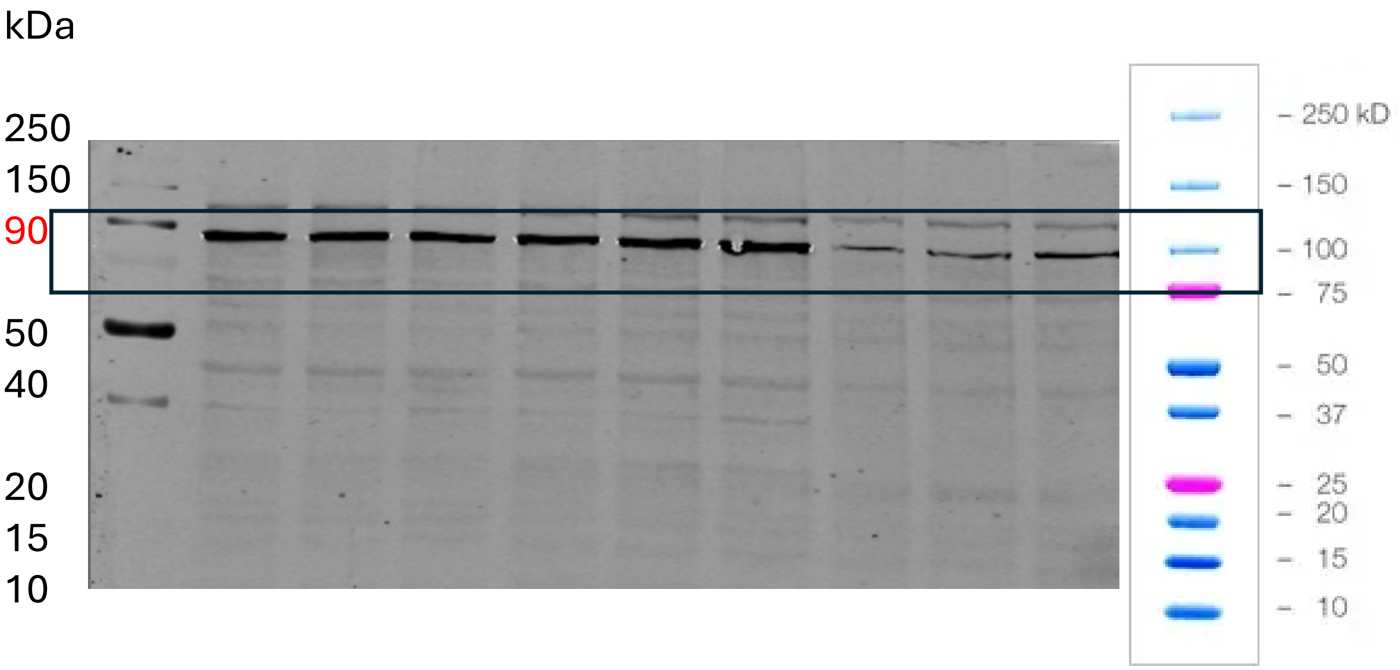

Gel membranes of Western blotting for **p-HSP90** (*in red* 90 kDa). On the right reference Ladder (Bio-rad Precision Plus Protein Dual Color Standards)

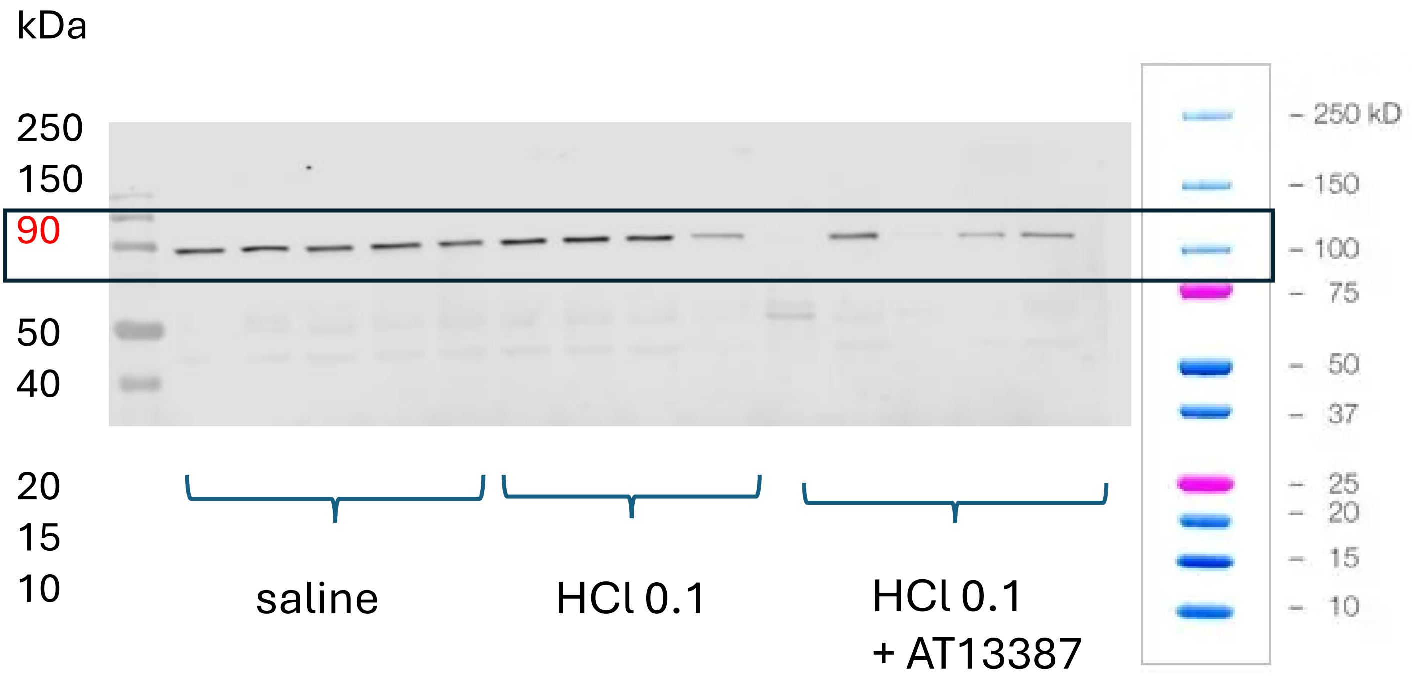

Gel membranes of Western blotting for **HSP90** (*in red* 90 kDa). On the right reference Ladder (Bio-rad Precision Plus Protein Dual Color Standards)

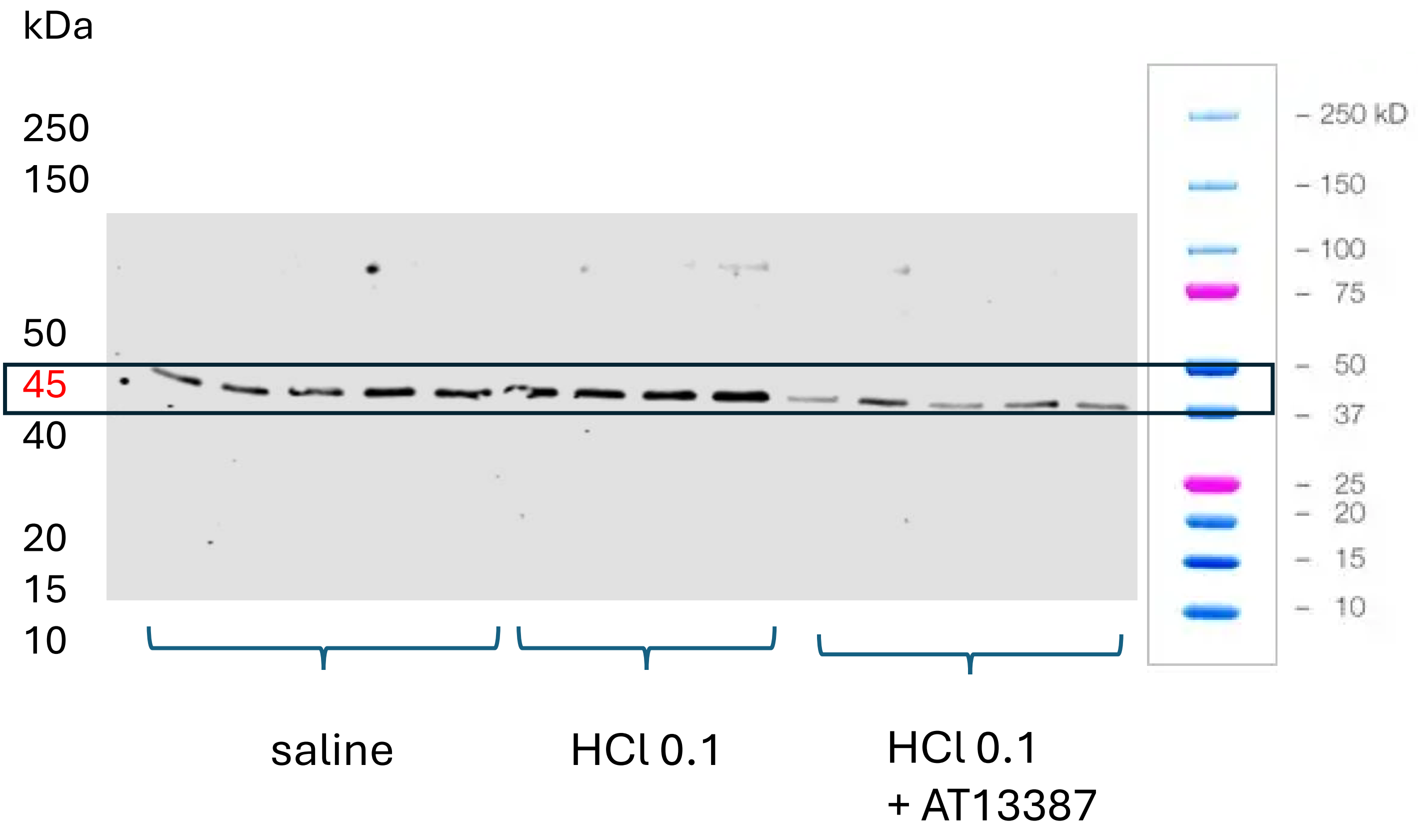

Gel membranes of Western blotting for **p-IKBa** (*in red* 45 kDa). On the right reference Ladder (Bio-rad Precision Plus Protein Dual Color Standards)

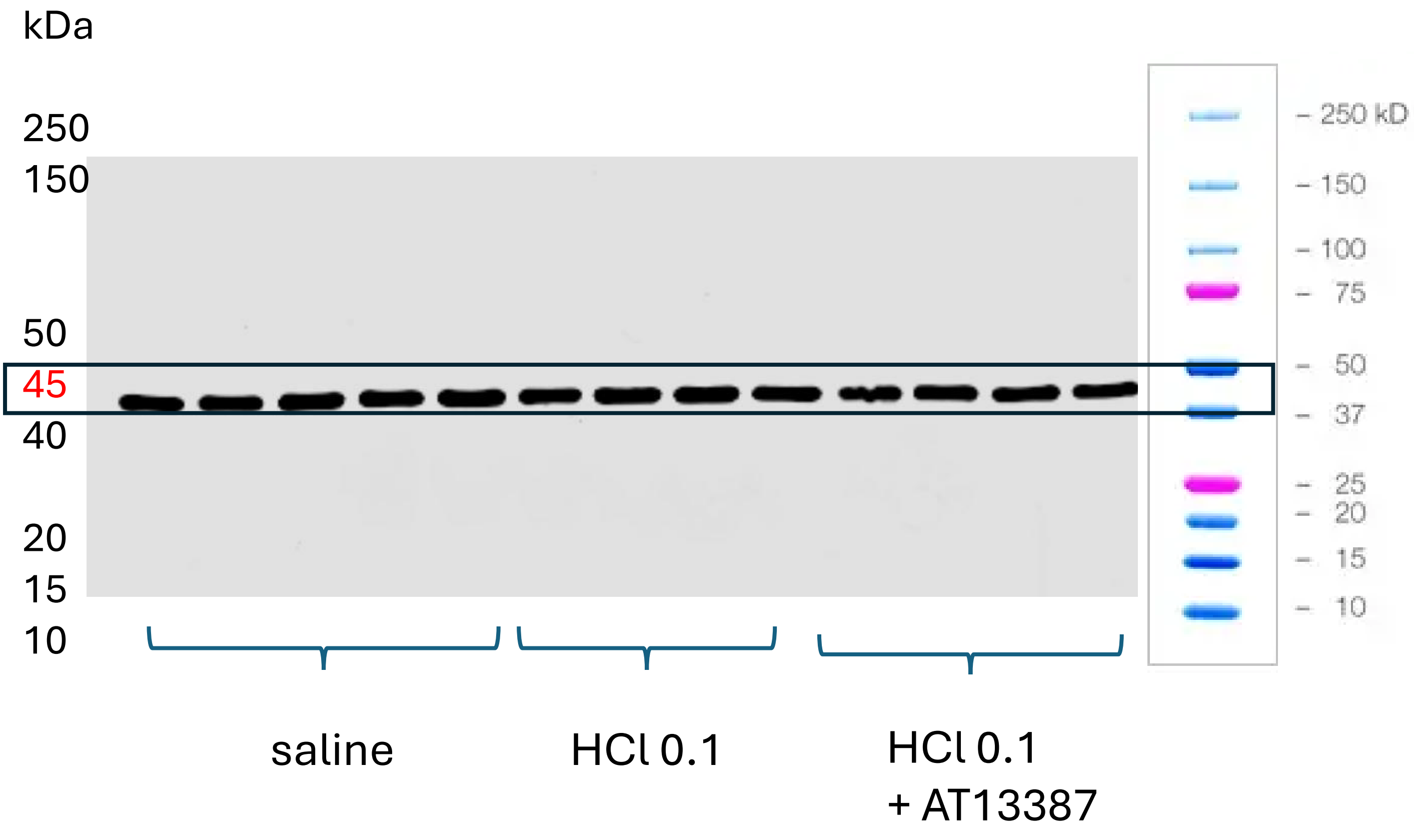

Gel membranes of Western blotting for **IKBa** (*in red* 45 kDa). On the right reference Ladder (Bio-rad Precision Plus Protein Dual Color Standards)

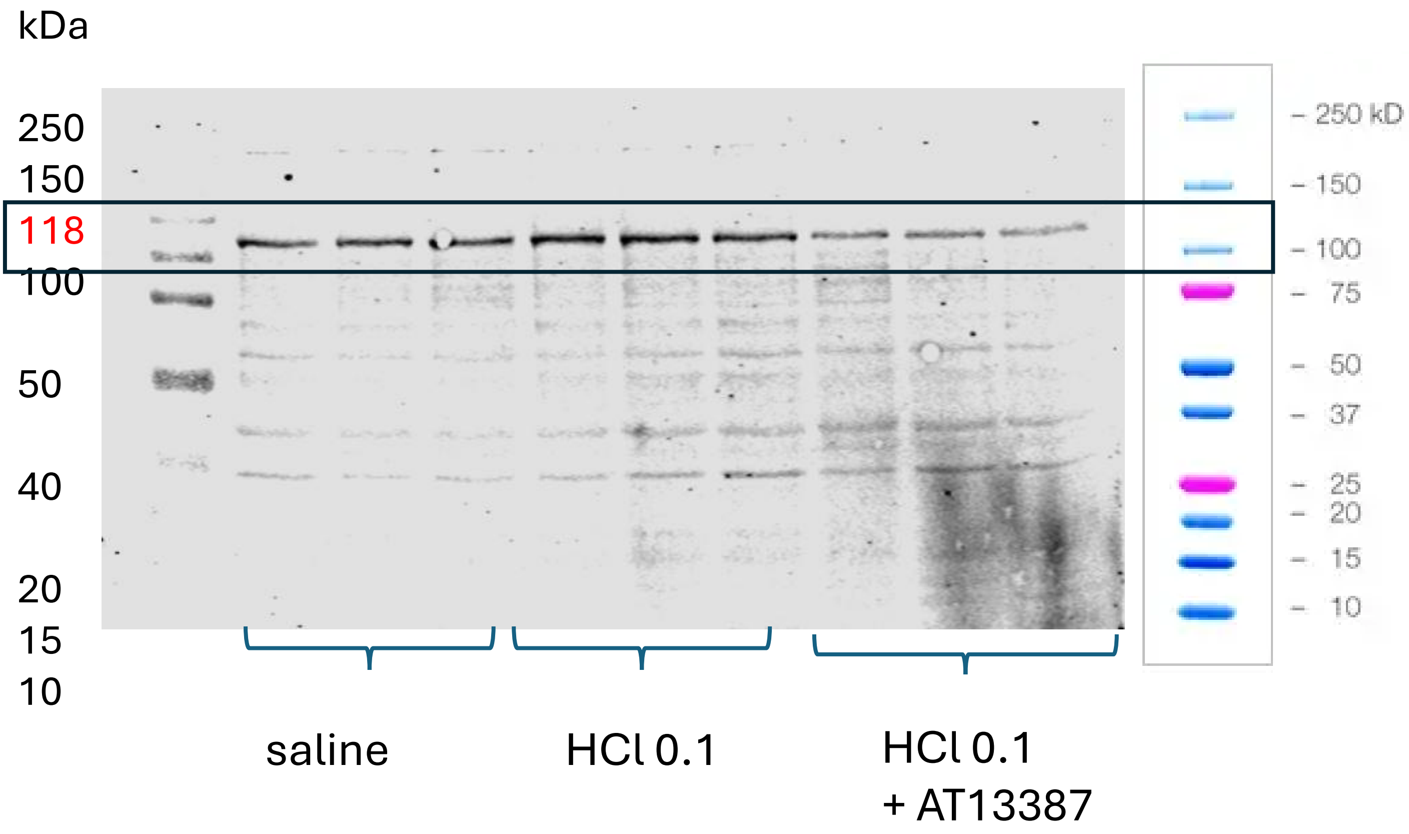

Gel membranes of Western blotting for **NLRP3** (*in red* 118 kDa). On the right reference Ladder (Bio-rad Precision Plus Protein Dual Color Standards)

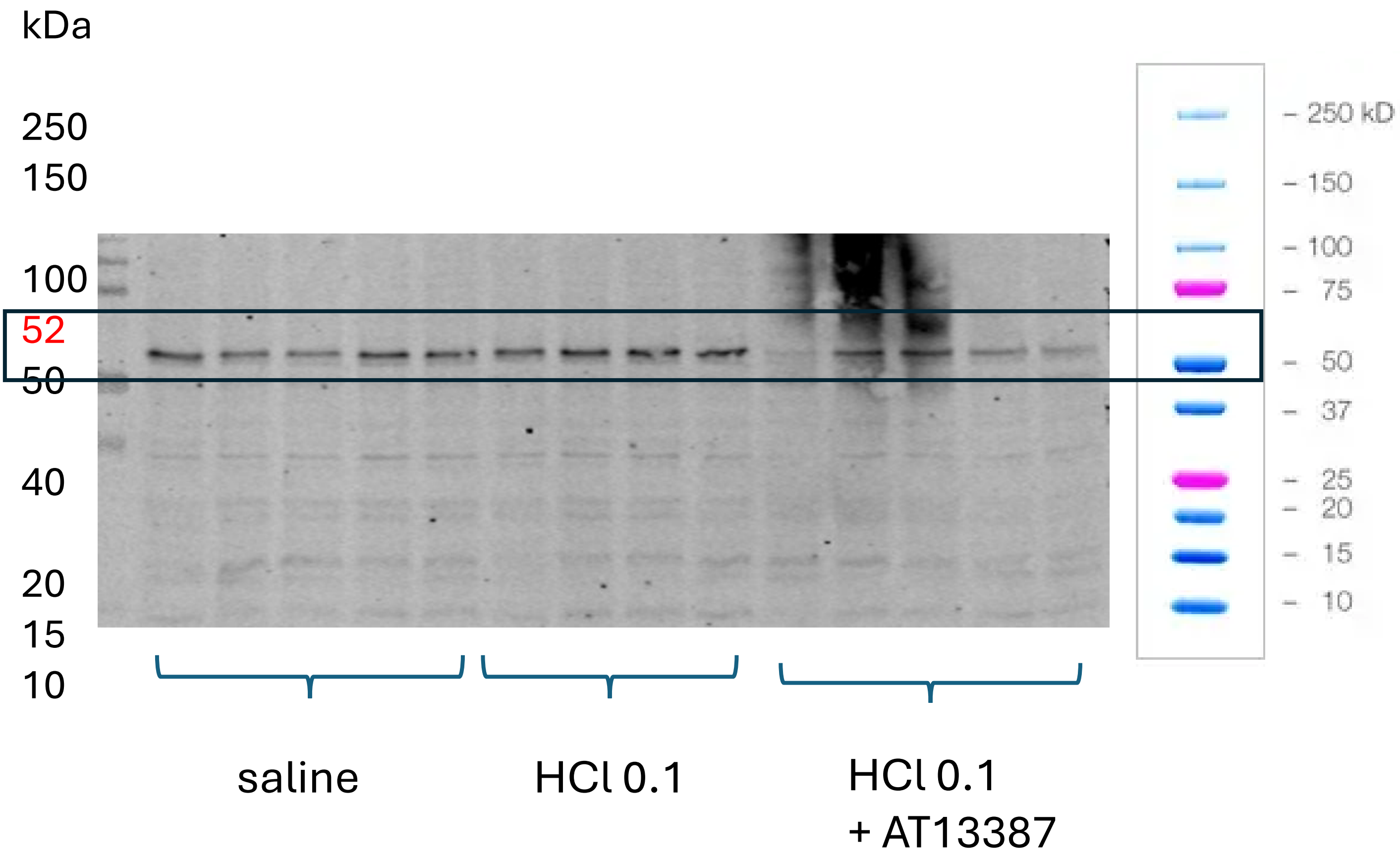

Gel membranes of Western blotting for **p-SMAD2** (*in red* 52 kDa). On the right reference Ladder (Bio-rad Precision Plus Protein Dual Color Standards)

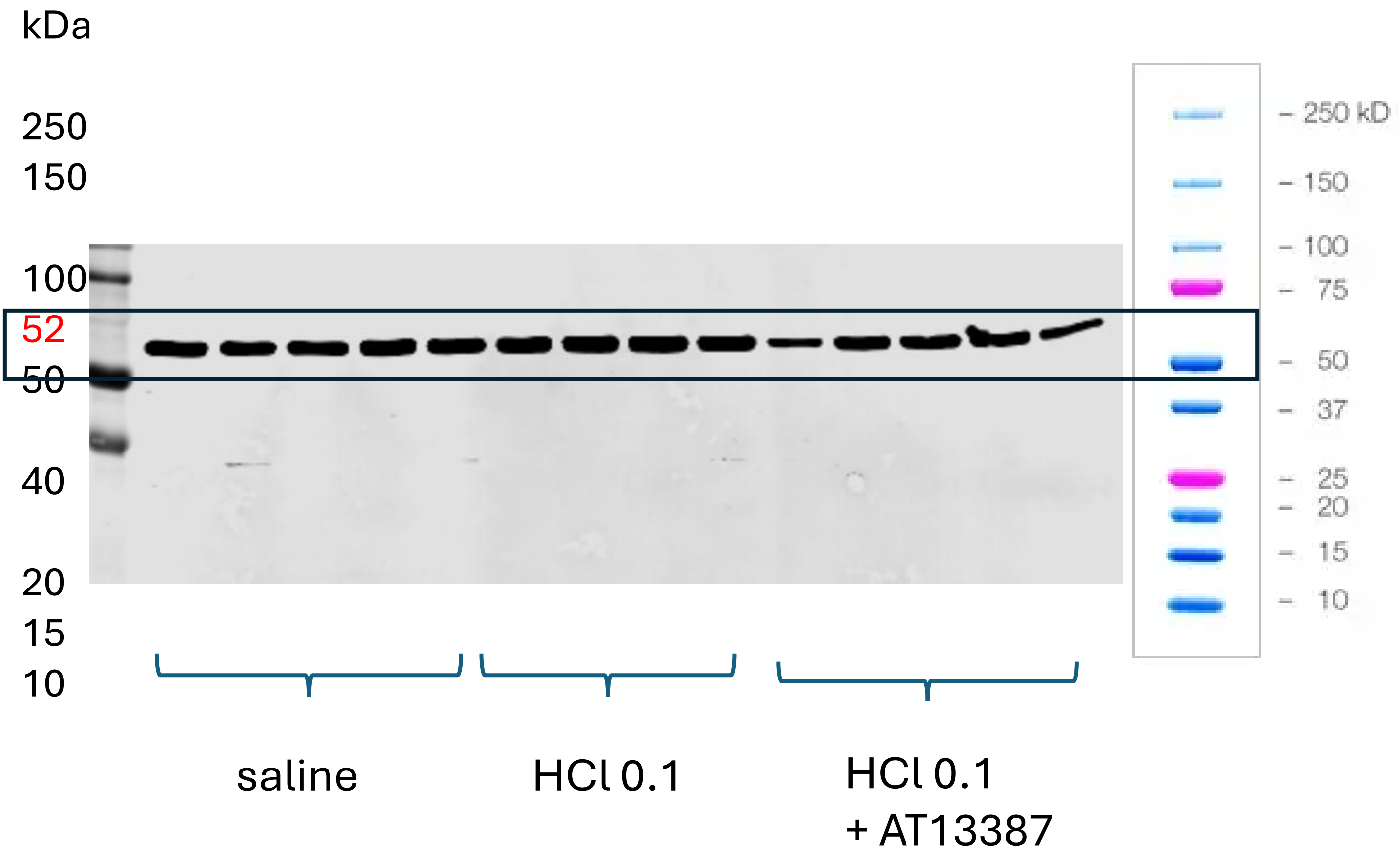

Gel membranes of Western blotting for **SMAD2** (*in red* 52 kDa). On the right reference Ladder (Bio-rad Precision Plus Protein Dual Color Standards)

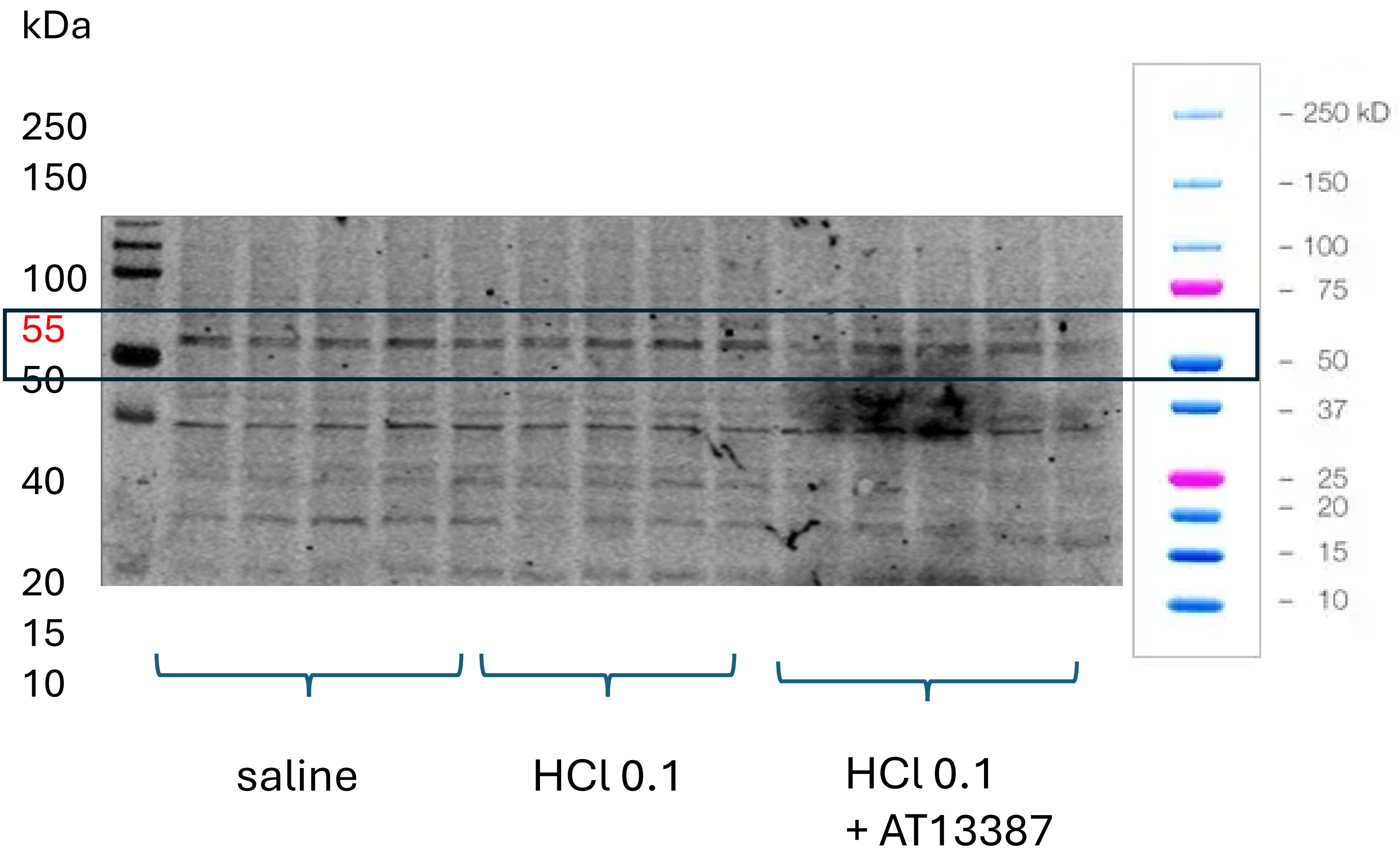

Gel membranes of Western blotting for **p-SMAD3** (*in red* 55 kDa). On the right reference Ladder (Bio-rad Precision Plus Protein Dual Color Standards)

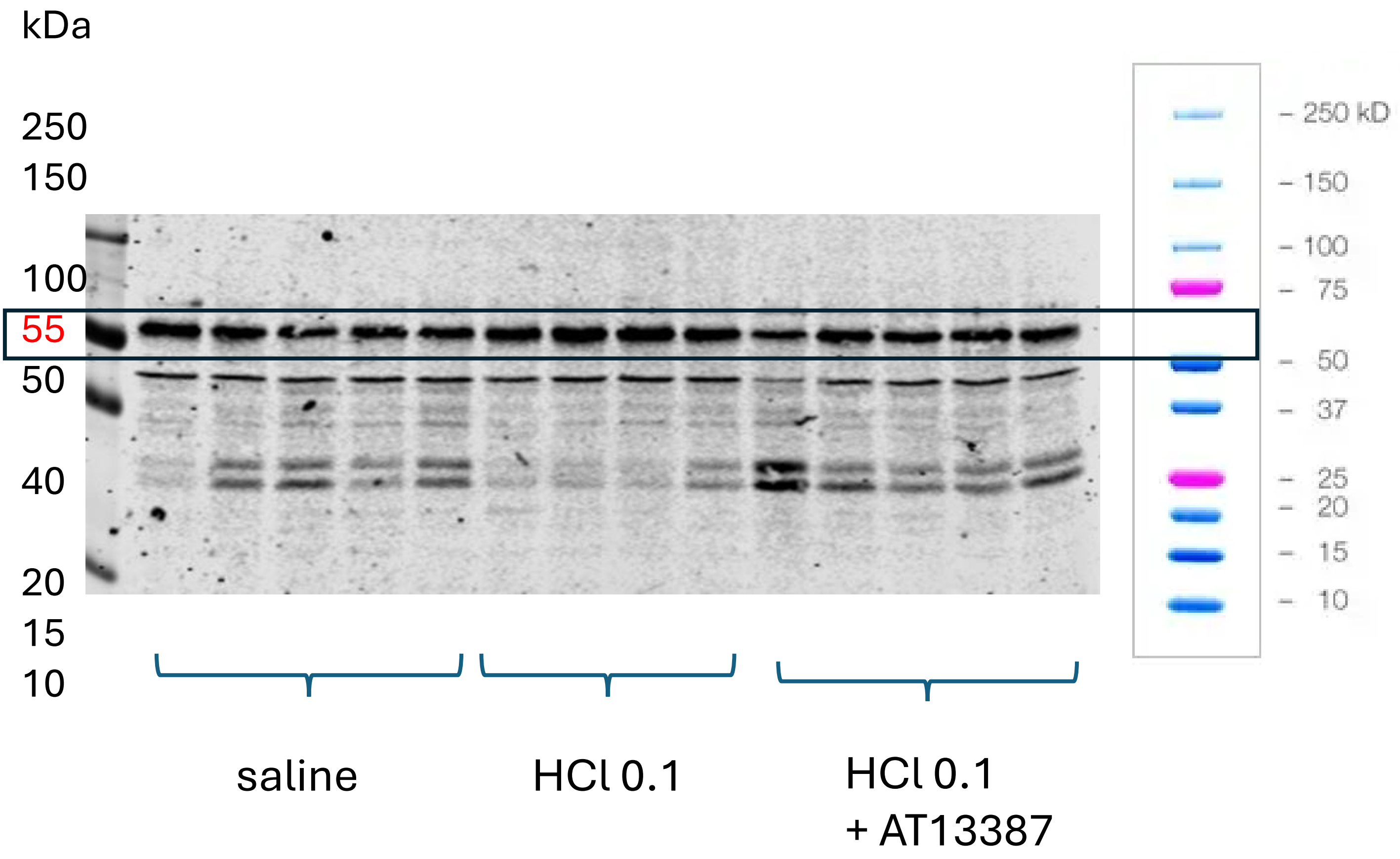

Gel membranes of Western blotting for **SMAD3** (*in red* 55 kDa). On the right reference Ladder (Bio-rad Precision Plus Protein Dual Color Standards)

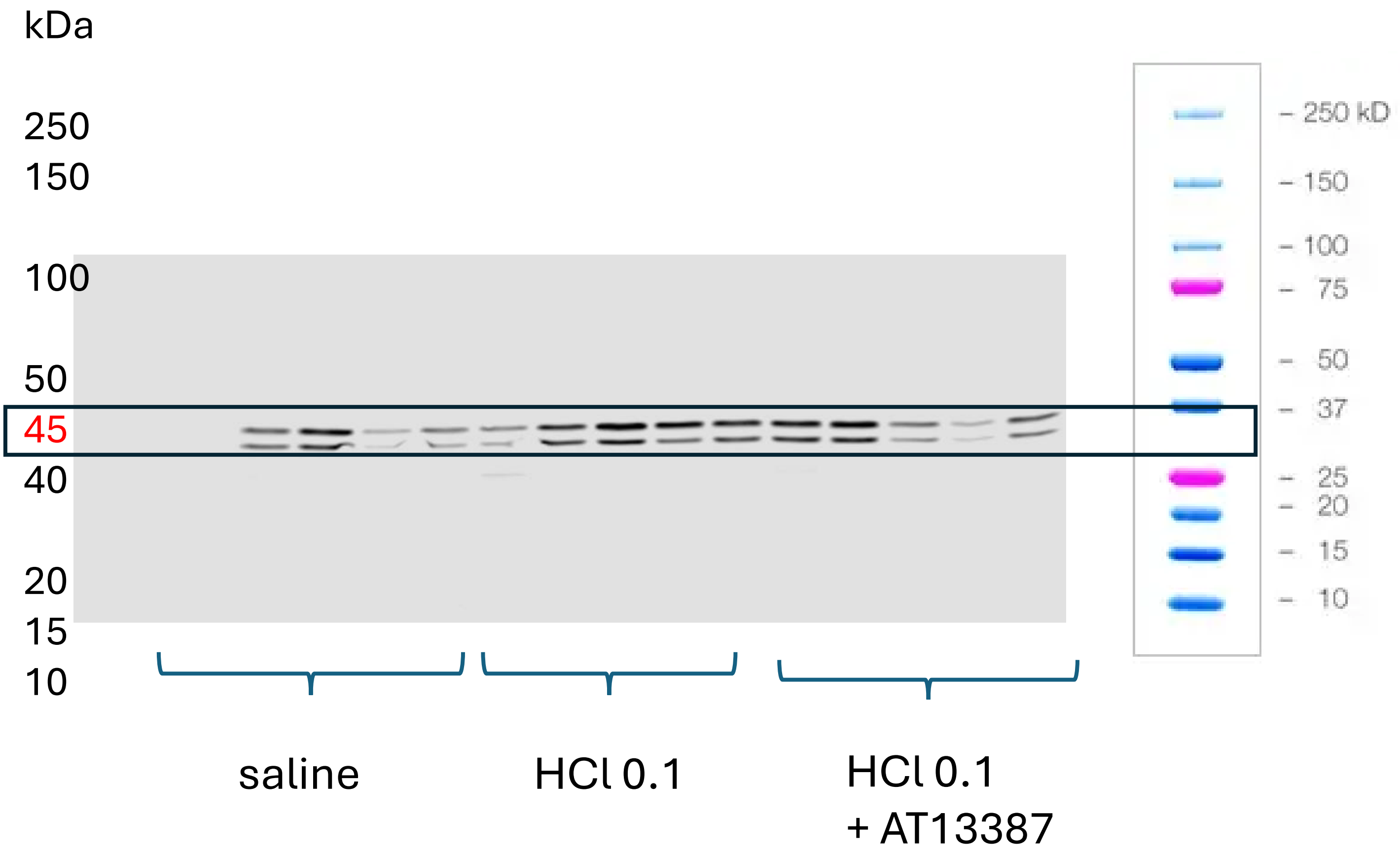

Gel membranes of Western blotting for **p-ERK** (*in red* 44 kDa). On the right reference Ladder (Bio-rad Precision Plus Protein Dual Color Standards)

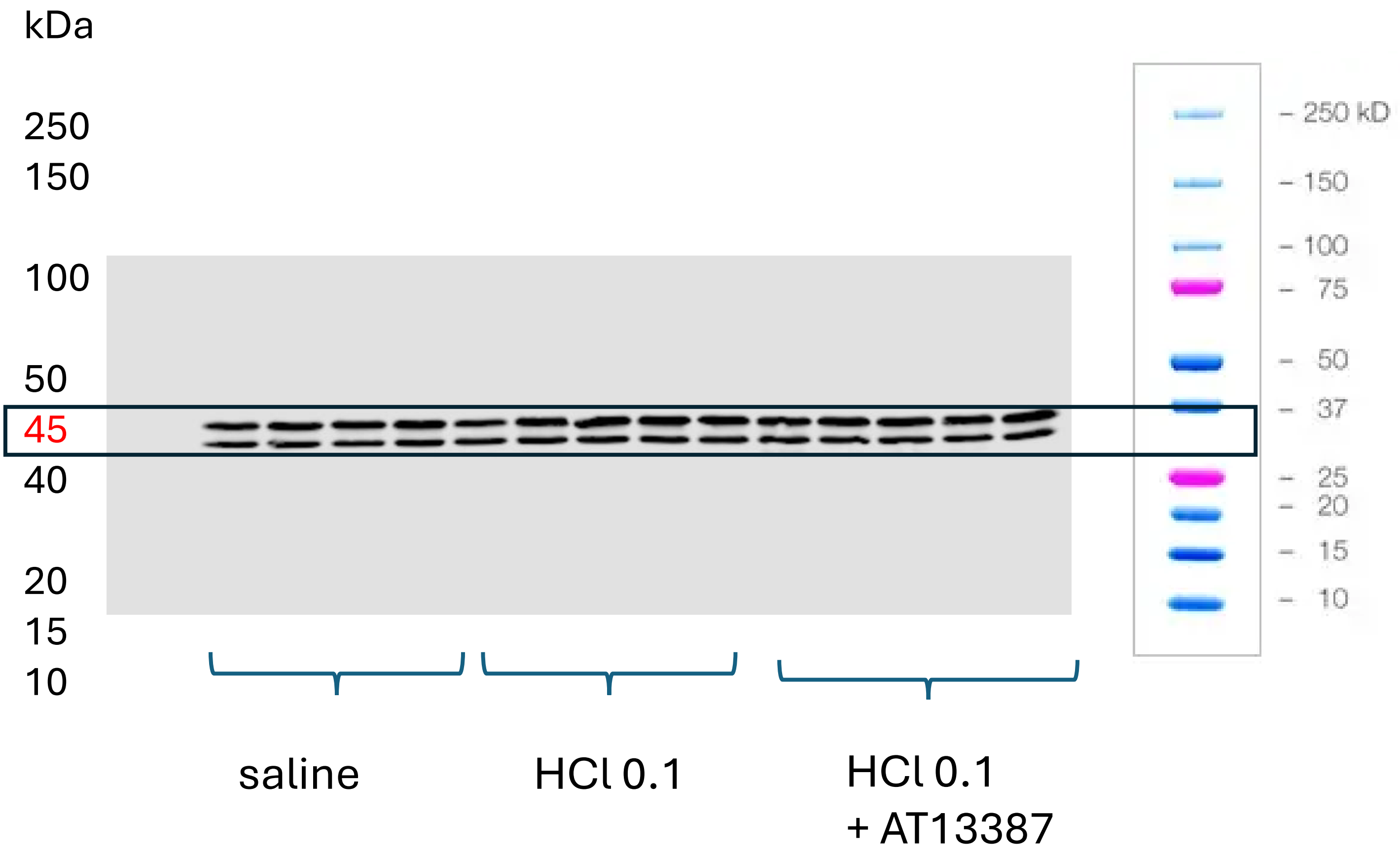

Gel membranes of Western blotting for **ERK** (*in red* 44 kDa). On the right reference Ladder (Bio-rad Precision Plus Protein Dual Color Standards)
